# Factors associated with uptake of influenza vaccine in people aged 50 to 64 years in Hong Kong: a case–control study

**DOI:** 10.1186/s12889-015-1990-0

**Published:** 2015-07-07

**Authors:** May PS Yeung, Stephen Kam-Cheung Ng, Edmond Tak Fai Tong, Stephen Sek-Kam Chan, Richard Coker

**Affiliations:** Department of Public Health and Policy, London School of Hygiene and Tropical Medicine, London, WC1E 7HT UK; General Practice, Unit A-C, 11/F, Power Industrial Building, 9-15 Wo Heung Street, Fotan, NT Hong Kong, China; School of Nursing, the Hong Kong Polytechnic University, Hong Kong, China; Statistics Section, Transport Department of the Hong Kong Government, 38/F, Immigration Tower, 7 Gloucester Road, Wan Chai, Hong Kong

**Keywords:** Influenza vaccine uptake, 50–64 years, Case–control study, Associated factors

## Abstract

**Background:**

In Hong Kong, people aged 50–64 years were added as a recommended priority group (recommended group) for influenza vaccination by the Department of Health (DH) starting from 2011/12 onwards. The coverage rate of influenza vaccination for this age group was suboptimal at 8.5 % in 2012/13. This study investigates the factors associated with the uptake of influenza vaccination among adults in Hong Kong aged 50–64 years.

**Methods:**

A case–control study was conducted in communities by street intercept interviews from 17 July to 15 August 2013. Cases were adults aged 50–64 years who had received the influenza vaccine in 2011/12 or 2012/13, while controls were the same as the cases, except they had not received the influenza vaccine in 2011/12 or 2012/13. Multiple logistic regression analysis was performed on the data to explore the associations between vaccination status and the variables.

**Results:**

Six hundred and four respondents in total were interviewed and included in the analysis. There were 193 cases (vaccinated) and 411 controls (non-vaccinated), with a case-to-control ratio of 1:2.1. The following were strongly associated with vaccination compared to other factors: ‘eligible for free government vaccine’ (OR6.38, 95 % CI, 3.43-11.87, *p* < 0.001); ‘willing to receive flu vaccination for free’ (OR4.84, 95 % CI, 2.13-11.03, *p* < 0.001); ‘perceived having severe or moderate symptoms when contracting flu’ (OR2.90, 95 % CI, 1.21-6.97, *p* = 0.02), and ‘convenient to reach a vaccination location’ (OR2.87, 95 % CI, 1.06-7.74, *p* = 0.04). The majority of the cases (80.8 %) and controls (93.9 %) were not aware that they belonged to a recommended group for influenza vaccination and most (>80 %) were willing to be vaccinated if it was free.

**Conclusions:**

Factors related to free and convenient vaccination, the perception of the severity of symptoms when contracting influenza had a comparatively strong association with influenza vaccination uptake amongst 50–64 year olds, compared to other factors.

## Background

Seasonal influenza vaccination (referred to as ‘influenza vaccination’, ‘vaccination’ or ‘vaccine’, below) remains an effective measure to protect individuals and communities from severe morbidity and mortality induced by influenza. To mitigate the disease burden of influenza, many developed countries recommend vaccination for high-risk groups. Some exceptions are the United States (US), Austria and Estonia, which have universally recommended people aged 6 months or above to receive influenza vaccination [1–3]. Few European countries, such as Belgium and Ireland, included those aged 50–64 years in their recommended groups [4].

Although the vaccine did not provide an overall economic benefit in some communities, it yielded significant health benefits by reducing severe complications from influenza [3, 5, 6]. Meta-analysis and literature reviews demonstrated that the influenza vaccine had a moderate effect in reducing the clinical symptoms of influenza in healthy people from 16 to 64 years [7, 8]. Many middle-aged adults have undiagnosed medical conditions, such as diabetes mellitus, and are at higher risk of severe influenza-related complications [9, 10].

In Hong Kong, people aged 50–64 years were added as a recommended priority group (recommended group) for influenza vaccination by the Department of Health (DH) starting from 2011/12 [11]. The major driver behind this inititiative was a real increase in influenza-attributed Intensive Care Unit (ICU) admissions and deaths among the middle-aged group in 2010/11, [7] plus an anticipated increase in the years to come when the influenza A(H1N1)pdm09 strain was predicted to circulate in the population.

After this new vaccination policy was launched, however, the vaccine was not well received and the vaccination coverage in this new target group was very low at 8.5 % [12]. No free or subsidised influenza vaccination service was provided by the Government to this group, except those who already belonged to the other free or subsidised recommended high-risk groups and those with financial difficulties, i.e., Comprehensive Social Security Assistance (CSSA) recipients. Healthy 50–64 year olds, without other risk indicators, had to pay if they wanted to be vaccinated. This study aimed to find out which factors were associated with the low uptake of influenza vaccination among people aged 50–64 years in Hong Kong.

## Methods

A survey was conducted in a community setting in Hong Kong from 17 July to 15 August 2013, following which a case–control analysis was used to investigate the study hypothesis. Street intercept interviews were undertaken in 6 districts (out of a total of 18 in the territory). Cases were (i) those who received the influenza vaccine in 2011/12 or 2012/13, i.e., from 1 September 2011 to 31 August 2013; (ii) aged 50–64 years in 2012–2013; and (iii) citizens who were resident in Hong Kong. Controls were the same as the cases in (ii) and (iii), except they had not received the influenza vaccine in 2011/12 or 2012/13 influenza seasons. Some controls had received the influenza vaccine before 1 September 2011. They were classified as control because they were not included as the recommended group in 2010/11 and before.

The sample size was calculated with a significance level of 0.05 (two-sided) and a power level of 0.80. The calculation of the sample size was done by the Fleiss formula for unmatched case–control studies with dichotomous exposure variables. A minimum sample size of 510 was required with a case-to-control ratio of 1:2 [13].

The interviewers were assigned a random time slot, covering weekdays, weekends, office and non-office hours. The questionnaire was conducted in summer 2013 before the next influenza vaccination season, which usually begins in September of each year. Primary data were collected by four trained research interviewers who were fluent in Chinese and English. The interviewers were stationed in areas of high pedestrian traffic, such as near underground train stations and shopping malls, during the assigned random time slot.

This research had been approved by the Human Subjects Ethics Sub-committee of the Hong Kong Polytechnic University and the Ethics Committee of the London School of Hygiene and Tropical Medicine. Before each interview, the interviewer would inform the respondent about the nature and purpose of the study and invited their voluntary participation. Interviewees were asked to respond only after informed consent was obtained. No incentive was given.

The hypothesis of this study was there were differences in associated factors (variables) between those Hong Kong residents aged 50–64 years who received the influenza vaccine in 2011/12 and 2012/13, and those who did not. The Null hypothesis assumes no such association.

The questionnaire was designed with reference to past vaccination questionnaires from health authorities [14, 15] and relevant studies [16–18]. The draft questionnaire was then sent for comment to a multi-disciplinary team, comprised of an infectious disease specialist, an epidemiologist and general practitioners. The questionnaire was in Chinese and English and had 38 questions including 11 on demographic data and 27 covering the factors (variables) to be examined.

Statistical analyses were performed using the software SAS 9.3. Categorical demographic data and variables were compared using the Pearson chi-square test, crude and adjusted odds ratios (ORs) with corresponding 95 % confidence intervals (CIs) and *p*-values. Multiple logistic regression analysis was performed. Any variables with *p* values <0.25 and those with important associations demonstrated in the literature were selected for regression analysis (backward stepwise regression algorithms). The regression model is a built-in formula in the SAS software. All statistical tests were two-tailed and variables were considered significant at a significance level of 0.05.

## Results

The study included 193 cases (vaccinated) and 411 controls (non-vaccinated), with a case to control ratio of 1:2.1. This sample size reached the required range in the sample size calculation. The average interview time was 7 min (standard deviation ±4 min) for each questionnaire, and the response rate was 41.7 %. During street intercept interviews, there were more non-vaccinated individuals (controls) than vaccinated ones (cases). After the required number of non-vaccinated was recruited, the excess approached by the interviewers were counted as non-responders. In total 210 man-hours were spent on the interviews.

### Demography

The differences between baseline demographic data of cases and controls were statistically insignificant regarding sex, ethics, education level, employment status, personal monthly income, current smoking and drinking status. The demography of cases and controls are shown in Table 1.

**Table 1:** Demography of the cases and controls

		Case (*n* = 193)		Control (*n* = 411)		Crude odd ratio (OR)			
		No.	%	No.	%	OR	95 % CI		*p*-value*
Average age (mean year) ± SD**		57.3 ± 4.8		56.3 ± 4.4		-	-		-
Sex	Male	73	37.8	159	38.7	1	ref***		ref
	Female	120	62.2	252	61.3	1.04	0.73	1.48	0.84
Age group	50 - 54	59	30.6	176	42.8	1	ref		ref
	55 - 59	63	32.6	119	29.0	1.58	1.03	2.41	0.04
	60 - 64	71	36.8	116	28.2	1.83	1.20	2.77	0.005
Ethnic	Chinese	193	100.0	410	99.8	-	-		-
Education	Primary or below	58	30.1	110	26.8	1	ref		ref
	Secondary	120	62.2	260	63.9	0.88	0.60	1.29	0.50
	Tertiary or above	15	7.8	37	9.1	0.77	0.39	1.52	0.46
Status	Retired	52	26.9	69	16.8	-	-		-
	Housewife	47	24.4	112	27.3	-	-		-
Employed	Yes	83	43.0	198	48.2	0.82	0.58	1.15	0.24
	No	110	57.0	213	51.8	1	ref		ref
Current smoker	Yes	16	8.3	20	4.9	1.77	0.89	3.49	0.11
	No	177	91.7	391	95.1	1	ref		ref

*p-value is the value of crude OR when compared to reference

**standard deviation

***ref is reference

**Table 2:** Comparing study variables between cases and controls by adjusted odds ratios*

	Cases (193)		Control (411)		Odds ratio (Adjusted)			
	Count	%	Count	%	value	95 % CI		*p*-value
**Knowledge**								
Knowing oneself to be in the recommended group for flu vaccine	37	19.6	25	6.1	**2.34**	**1.23**	**4.44**	**0.01**
Knowing flu vaccine provides 70–90 % protection in healthy adults	89	46.6	140	34.1	**1.86**	**1.12**	**3.10**	**0.02**
Knowing about the Government Vaccination Programme	128	68.0	219	53.5	1.09	0.68	1.75	0.72
Knowing flu vaccine reduces flu complications and related hospitalisation	180	97.8	366	93.4	0.89	0.25	3.19	0.86
**Needs**								
Live with children < 6 years or elderly >65 years	53	27.5	85	20.7	1.23	0.74	2.04	0.43
Presence of chronic disease(s)	78	40.4	111	27.1	1.13	0.65	1.96	0.67
Visited doctors in the past 3 months	92	47.7	141	34.3	1.12	0.67	1.87	0.68
**Behaviour**								
Current smoker	16	8.3	20	4.9	0.72	0.27	1.92	0.52
Current drinker	34	17.6	80	19.5	-	-	-	-
Belief & perception								
Perceived having severe or moderate symptoms when contracting flu	17	8.8	15	3.6	**2.90**	**1.21**	**6.97**	**0.02**
Perceived flu vaccine to be safe	183	98.4	342	89.8	3.99	0.78	20.41	0.10
Believed flu vaccine has additional benefits other than flu protection	181	93.8	356	87.3	1.36	0.58	3.21	0.48
**Health-care system**								
Eligible for free government vaccine	45	23.6	21	5.1	**6.38**	**3.43**	**11.87**	**<0.001**
Willing to receive flu vaccination for free	184	95.8	309	75.7	**4.84**	**2.13**	**11.03**	**<0.001**
Convenient to reach a vaccination location	187	96.9	370	90.0	**2.87**	**1.06**	**7.74**	**0.04**
Prefer public clinic for injection	105	54.7	291	71.0	**0.35**	**0.22**	**0.55**	**<0.001**
Will respond to Government telephone reminder service on flu shot	105	54.4	160	39.6	0.84	0.51	1.38	0.49
**Advice**								
Accept advice from health professionals	183	94.8	333	81.2	**2.67**	**1.19**	**5.99**	**0.02**
Had family member receive flu vaccine	76	41.8	74	18.3	**2.47**	**1.54**	**3.95**	**<0.001**
Accept advice from relatives and friends	86	44.8	101	24.6	1.47	0.84	2.56	0.18
**External factors**								
Will receive flu vaccine when there is an epidemic	181	94.8	327	81.3	**2.40**	**1.07**	**5.37**	**0.03**

*All study variables in this table are binary categorical variables (yes/no) with the ‘no’ category being used as the reference group for calculating ORs. Statistically significant odds ratios (adjusted) are bold.

There was no apparent discrepancy in the sample and the target population. One exception was in the sampled respondents; there were proportionally higher numbers of females than males (M:F = 1:1.6), while the overall ratio in the target community was 1:1. Other demographic parameters of the sampled population, such as the age proportion between groups, education level, ethnicity, and the percentage of those in employment, were comparable to the target population (i.e., Hong Kong general population aged 50–64 years).

The majority of the respondents were Chinese, and there were more female than male respondents (38.4 % vs 61.6 %). Most (86.5 %) of those who were in employment were aged 59 years or below. Overall, half of the respondents (51.5 %) had no income. One in four (26.3 %) was a housewife and one fifth (20.0 %) was retired. The majority of them (71.5 %) had received at least 9 years of education up to secondary level.

### Health knowledge related to influenza vaccine

The majority of all the cases (80.8 %) and controls (93.9 %) were not aware that the health authority had recommended vaccination against influenza. However, the cases were more aware of the recommendation for influenza vaccination than the controls, (OR2.34, 95 % CI 1.23-4.44, *p* = 0.009). There were health knowledge differences between the cases and controls in all the questions asked on knowledge, including government vaccination services, vaccine reduction in influenza-related hospital admission, and vaccine protection for healthy adults. However, these associations were statistically insignificant after the OR was adjusted (Table 2). 

### Health needs

When compared to the controls, more of the cases had chronic diseases; more frequently ‘visited doctors in the past 3 months’ and ‘lived with children below 6 years or elders above 65 years’. However, none of these associations was statistically significant after the OR was adjusted.

### Health behaviours

There was no association between vaccination and smoking/drinking. Most cases (85.4 %) stated that they were likely or very likely to receive the vaccine in 2013/14, compared to only 29.4 % among the controls. This implies those who had previous vaccinations in 2010/11 and 2011/12 would choose to be vaccinated again in the future.

### Health belief and perception

In general, more cases perceived there to be a higher risk of contracting influenza in the next 12 months and/or having severe influenza or moderate symptoms when compared to the controls. Vaccination was perceived more positively by the cases than the controls. More cases than controls ‘perceived flu vaccine to be safe’ and ‘believed flu vaccine has additional benefits other than flu protection’. However, the only statistically significant variable was ‘perception of severe or moderate symptoms when contracting flu’ with an OR of 2.90 (95 % CI 1.21-6.97, *p* = 0.02).

### Health-care system

There was an association between ‘eligible for free government vaccine’ and vaccination (OR6.38, 95 % CI 3.43-11.87, *p* < 0.001). When compared with controls, more cases were ‘willing to receive flu vaccination for free’ (OR4.84, 95 % CI 2.13-11.03, *p* < 0.001). Ninety-five percent (95.8 %) of the cases and 75 % (75.7 %) were ‘willing to receive flu vaccination for free’. Cases had a higher likelihood of being able to access a convenient location for vaccination (OR2.87, 95 % CI 1.06-7.74, *p* = 0.04). Fewer cases preferred to go to a public clinic for an injection (OR0.35, 95 % CI 0.22-0.55, *p* < 0.001). There were no associations between differences in response to the government telephone reminder service for vaccination, if there was one.

### Advice

In respect of vaccination, the cases were more heavily influenced by others’ opinions and actions than were the controls. When compared, more cases would ‘accept advice from health professionals’ (OR2.67, 95 % CI 1.19-5.99, *p* = 0.02); and ‘family members who had received the flu vaccine’ than the controls (OR2.47, 95 % CI 1.54-3.95, *p* < 0.001).

### External factors

External factors refer to unpredictable environmental factors, such as the occurrence of disease epidemics like Severe Acute Respiratory Syndrome (SARS) or pandemic influenza. High percentages of both cases (94.8 %) and controls (81.3 %) would receive a vaccine when there was a disease epidemic. When compared, more cases would receive a vaccine during an epidemic and the OR was 2.40 (95 % CI 1.07-5.37, *p* = 0.03).

### Additional information

Among the controls (i.e., never received vaccination or received vaccine on or before 2009/10), 25.6 % of them had previously received the vaccine. The following were common reasons given by the controls for not receiving a vaccine: considered vaccination unnecessary (70.8 %); believed they were not in a high-risk group (37.0 %); and concerns about side effects of vaccination (19.0 %). Of the controls that had previously been vaccinated, 59 % had received the vaccine at a public clinic.

A subgroup analysis was performed on those who received influenza vaccination but did not know they were recommended group by the Department of Health (DH). There were 193 cases (who were vaccinated) and among them 37 answered yes to “knowing oneself to be in the recommended group for flu vaccine” and 156 answered no. In these 156 people the five commonest reasons for vaccination were: advice from healthcare professionals (58.8 %), vaccine was useful in protect oneself against flu (43.1 %), flu shot had additional benefits, e.g. protect family member (23.5 %), perception of not having very good or good health (16.3 %) and eligible for free government vaccine (10.46 %). More than half (53 %) of these 156 people received their influenza vaccine at Government public clinics, and most of the remaining (41 %) at private general practitioners.

## Discussion

This is a case–control study with vaccination status as the ‘outcome’ and personal or external environmental factors as ‘exposures’. A case–control study design was chosen because of a low prevalence of eligible cases. A street intercept interview method enabled the interviewers to screen and approach a larger number of people, according to the outward appearance of their age. This probably lowered the rejection rate and enabled a greater control in completing the questionnaire. It was estimated that a larger number of people would have had to be approached should a telephone or postage survey been used. The low response rate (41.7 %) was attributable to the difficulty in finding cases, as the excess controls approached by the interviewers were counted as non-responders. Moreover, the interviews were conducted in summer time when the street temperature was >30 °C, the streets were crowded and no incentive was offered.

Multi-dimensional factors have contributed to people’s choice of whether or not to receive vaccination. These factors comprise of social, environmental and economic dynamics in a specific context. The factors were put in a multiple logistic regression model and statistically adjusted for age, employment status, in receipt of social security, and all independent variables. Before statistical adjustment, most of these factors had statistically significant crude odds ratios. The variables affected each other and many became non-significant after adjustment. There would be a confounding effect between variables.

### Discussion on study results

The majority of the cases (80.8 %) and controls (93.9 %) were not aware that they were in a group recommended by the health authority to receive influenza vaccination. Among the controls, a higher percentage (71 %) deemed vaccination to be ‘unnecessary’. This revealed a failure of DH and health professionals in communicating the message that ‘vaccination is recommended’ to this age group. Given that there was an association between ‘knowing oneself to be in the recommended group for flu vaccine’ and vaccination, better communication of the risks might have improved the vaccination rate. A health promotion strategy on empowerment and enhancement of knowledge on this issue needs to be planned and supported by health-care policy.

Studies suggested that previous influenza vaccination was a predictor for subsequent vaccination (OR 1.62-5.40) [19–22]. However, past behaviour does not provide an insight into the reasons why a person chooses to be vaccinated.

The vaccination coverage rate is price sensitive. This was demonstrated in this study and in countries which provided vaccine reimbursements to users [23, 24]. To receive influenza vaccination, most (95 %) people aged 50–64 years in the general Hong Kong population had to pay out-of-pocket. In this study, the odds of the cases being ‘eligible for free government vaccine’ were 6.4 times the controls. Among the cases, half (52 %) of them attended a private clinic or hospital and paid the vaccination fee. Many study cases and controls expressed they were willing to receive the vaccine if it was free or subsidised. Such a vaccination service could possibly increase the vaccination rate.

There was only a mild association between chronic disease(s) and vaccination and the association was insignificant after the OR was adjusted (OR1.13, 95 % CI 0.65-1.96, *p* = 0.67). This result contradicted the findings of many studies that indicated that the presence of chronic diseases was one of the most persistent factors associated with vaccination [19, 20, 25–30].

‘Accept advice by health professional’ was moderately associated with vaccination (OR2.67, 95 % CI 1.19-5.99, *p* = 0.02). Several other studies have shown that doctors’ and health professionals’ advice was associated with influenza vaccination [17, 31]. Health professionals had a duty to recommend vaccination to high-risk groups in order to protect them from influenza and severe complications.

‘Had family member received flu vaccine’ was associated with people’s uptake of the vaccination, but ‘accept advice from relatives and friends’ was not. In Japan, advice from health professionals, family and/or close friends was strongly associated [17]. In the USA and other western countries, advice from family and/or close friends was not a significant factor in acceptance of influenza vaccination [32, 33]. This could possibly be due to the differences in cultural backgrounds between individuals in these countries.

This study showed no association between vaccination and smoking and drinking. It is uncertain whether people were consistent in their health behaviours. Studies have proven that smoking is not associated with vaccination [21, 34]. No data was found on other health behaviours, such as drinking or frequent exercise, having a link to vaccination.

Given past experiences of infectious disease epidemics in Hong Kong, people may be more inclined to receive vaccination to protect themselves in anticipation of the occurrence of a disease epidemic such as SARS or swine influenza.

### Discussion on vaccination policy

Previous research has suggested that newly issued recommendations are not quickly embraced by the majority of citizens. In the US, government National Health Interview Survey data did not show a marked increase in vaccination rates among adults aged 19–49 and 50–64 years after the US Advisory Committee on Immunization Practices expanded its recommendations to these subgroups in 2000 and 2010, respectively [31, 35].

This vaccination policy limited the government vaccination free service to those suffering economic hardship and chronic diseases among 50–64 year-olds. Although the price of receiving an influenza vaccination constitutes a minute percentage of monthly income, this does not necessarily mean socio-economically deprived groups who are ineligible for free vaccination would be willing to pay for the vaccine. Subsidised vaccination would attract those who are willing to pay at a discounted price. Health providers could be engaged, with or without incentives, to promote the benefit of vaccination. In addition, DH should consider health promotion messages addressing factors with strong associations to encourage payment by the individual. These factors included ‘the perception of having severe or moderate symptoms when contracting flu’, ‘knowledge of being in the recommended group for flu vaccine’ and ‘good vaccine protection for healthy adults’.

### Discussion on study strength and limitations

A case–control design enabled the measurement of many different exposures at once and for the combined effects of exposures to be examined. In addition, data were collected within a short time-frame. One of the important limitations of this case–control was the temporal sequence and reverse causality. It is difficult to interpret the time sequence of the exposures and the outcomes. For example, it is uncertain whether perception of the safety of the influenza vaccine was a cause or a consequence of vaccination. Other limitations of this case–control include the information and recall bias of the respondents, and the inability to estimate the coverage of vaccination in this age band.

One limitation of using the street-intercept method would be the possibility that the interviewers approached those who looked 50–64 years and, potentially missed a number of younger and older looking individuals; the extent of this bias is difficult to assess. Another bias would be due to the sampling of respondents from different locations, e.g., on public and private estates, in train stations and shopping malls. A comparison of the demographic characteristics of the samples collected in different locations, and those of the relevant population, would be useful to identify potential bias.

The study results have important implications for the general population aged 50–64 years in Hong Kong. There would be considerable differences between cultures, beliefs, norms and external environments - such as health systems and service provision - which have to be taken into consideration when applying the results to other populations. Further studies on the local vaccination policy and the views of health professionals would provide a comprehensive account of the low vaccination coverage in this age group.

## Conclusions

Factors related to free and convenient vaccination, perception of the severity of symptoms when contracting influenza had a comparatively strong association with influenza vaccination uptake among 50–64 year olds, compared to other factors.
